# The integrated disease surveillance and response system in northern Ghana: challenges to the core and support functions

**DOI:** 10.1186/s12913-015-0960-7

**Published:** 2015-07-28

**Authors:** Martin N Adokiya, John K Awoonor-Williams, Claudia Beiersmann, Olaf Müller

**Affiliations:** Institute of Public Health, University of Heidelberg, INF 324, D-69120 Heidelberg, Germany; School of Medicine & Health Sciences, University for Development Studies, Box TL 1350, Tamale, Ghana; Regional Health Directorate, Ghana Health Service, Upper East Region, Bolgatanga, Ghana, Swiss Tropical and Public Health Institute, Switzerland, and University of Basel, Basel, Switzerland

**Keywords:** Disease surveillance, Core and support functions, Health information system, Ghana

## Abstract

**Background:**

The integrated disease surveillance and response (IDSR) strategy was adopted in Ghana over a decade ago, yet gaps still remain in its proper functioning. The objective of this study was to assess the core and support functions of the IDSR system at the periphery level of the health system in northern Ghana.

**Methods:**

A qualitative study has been conducted among 18 key informants in two districts of Upper East Region. The respondents were from 9 health facilities considered representative of the health system (public, private and mission). A semi-structured questionnaire with focus on core and support functions (e.g. case detection, confirmation, reporting, analysis, investigation, response, training, supervision and resources) of the IDSR system was administered to the respondents. The responses were recorded according to specific themes.

**Results:**

The majority (7/9) of health facilities had designated disease surveillance officers. Some informants were of the opinion that the core and support functions of the IDSR system had improved over time. In particular, mobile phone reporting was mentioned to have made IDSR report submission easier. However, none of the health facilities had copies of the IDSR Technical Guidelines for standard case definitions, laboratories were ill-equipped, supervision was largely absent and feedback occurred rather irregular. Informants also reported, that the community perceived diagnostic testing at the health facilities to be unreliable (e.g. tuberculosis, Human Immunodeficiency Virus). In addition, disease surveillance activities were of low priority for nurses, doctors, administrators and laboratory workers.

**Conclusions:**

Although the IDSR system was associated with some benefits to the system such as reporting and accessibility of surveillance reports, there remain major challenges to the functioning and the quality of IDSR in Ghana. Disease surveillance needs to be much strengthened in West Africa to cope with outbreaks such as the recent Ebola epidemic.

## Background

Surveillance is the ongoing systematic collection, analysis and interpretation of health data essential for planning, implementation, and evaluation of public health practice, closely integrated with timely dissemination of these data to those who need to know [1, 2]. In spite of increased efforts in strengthening health systems, developing countries still have a long way to go to achieve well-functioning health systems. In particular the International Health Regulations (IHR 2005), which require Member States to strengthen their existing capacity for disease surveillance and response using the IDSR strategy, are still not well implemented [3–5]. As a result, data of poor quality are used for planning and decision-making in many developing countries [6]. These problems are aggravated by disease-specific programs continuing to implement separate surveillance systems which lead to a proliferation of indicators and overburdening of the health workforce [7].

In the 1990s, a number of disease outbreaks (e.g. yellow fever, cholera, meningococcal meningitis and Ebola and Marburg haemorrhagic fevers) occurred in Africa due to emerging and re-emerging pathogens [4]. As a result, the World Health Organization Regional Office for Africa (WHO-AFRO) and other partners were asked by the various ministries of health to develop strategies that would enable countries to respond adequately to these challenges including detection and confirmation of diseases in time and overall strengthening of surveillance capacities [4]. In 1998, WHO-AFRO adopted the Integrated Disease Surveillance and Response (IDSR) strategy for its member countries as a comprehensive public health strategy [5, 8–11]. The goal of the IDSR strategy is to build member countries capacity to detect, report and effectively respond to priority diseases as well as to integrate multiple existing vertical surveillance systems, and linking laboratory and other data sources for public health action [9, 12-14]. The IDSR strategy particularly focuses on the district level of the health systems [13].

**Fig. 1:**
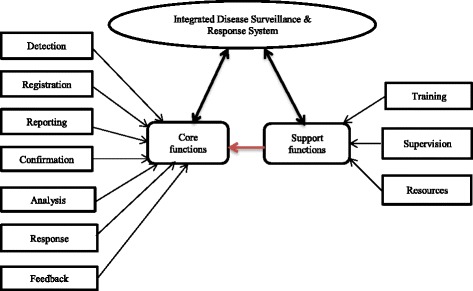
Key concepts of IDSR. Source: modified from conceptual framework of public health surveillance [14]

Ghana has experienced severe outbreaks of cholera, meningococcal meningitis and yellow fever in the years 1996/97 [15]. In 2002, Ghana adopted the IDSR strategy and implemented it nationwide to improve the disease surveillance system. The strategy has since seen a decade of implementation and the number of diseases required for reporting increased from 23 in 2002 to 43 in 2011 [16, 17]. This increase was due to several epidemiological considerations including social, economic, and environmental changes [16]. In addition, the increased number of diseases reflects the added requirements of the IHR (2005) [4]. With these requirements, weak health systems with inadequate infrastructure may not be effective in disease surveillance. Besides, the limited human resources in the health system may further be overburdened with data processing and reporting. Recently, the District Health Information Management System II (DHIMS2) was introduced as an internet-based system for nationwide reporting. This requires IDSR data to be reported through the DHIMS2 with the overall goal of reducing the reporting burden in primary health care settings and to improve data quality and reliability [18]. The objective of this study was to assess the core and support functions of the IDSR strategy at the periphery level of the health systems in northern Ghana (Fig. 1).

### Ghana health care system

**Fig. 2:**
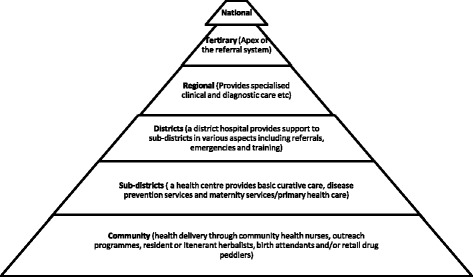
Organization of the health system in Ghana. Source: adopted from Ghana health service [20]

Ghana’s health care system is organized in a three tier system (i.e. district, region and national). This is further structured into five levels; national, regional, district, sub-district and community. The smallest unit of the health system is the Community-based Health Planning and Services (CHPS), which is responsible for the provision of community level health activities including treatment of minor ailments, home-visits, community outreaches, education and health promotion [19]. Primary Health Care (PHC) is delivered by the district health system and entails all institutions including CHPS, health centres, clinics and hospitals. The health centre is mainly responsible for providing clinical, public health and maternity services and uses a combination of facility-based services, regular outreaches and mass campaigns in close collaboration with communities, community institutions, leaders and community-based health workers. The district hospital serves as the first referral point in the PHC system in the country [19, 20] where clinical, surgical, laboratory and maternity services are provided (Fig. 2).

The definition of *periphery* health system in this study includes Community-Based Surveillance Volunteers (CBSV), Community-based Health Planning and Service (CHPS), health centres, mission and private clinics and the district hospital.

## Methods

### Study setting

**Fig. 3:**
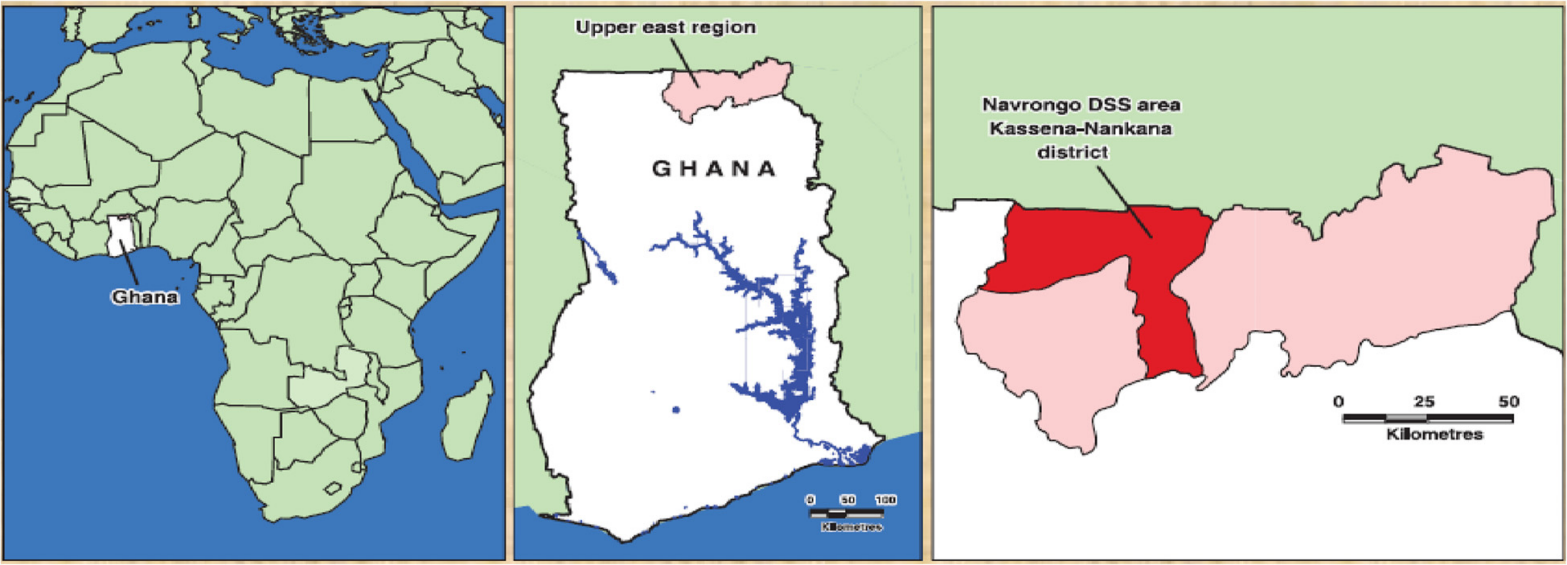
Location of Ghana in Africa, the Upper East Region and the Kassena-Nankana Districts. Source: Profile of the Navrongo Health and Demographic Surveillance System [25]

Ghana is situated in West Africa and bordered by Ivory Coast to the west, Burkina Faso to the north, Togo to the east, and the Atlantic Ocean to the south (Fig. 3). Administratively, the country is composed of 10 administrative regions and 216 districts. The Kassena-Nankana districts (Kassena-Nankana Municipal and Kassena-Nankana West) are situated in the Upper East Region (UER) [21], one of the poorest regions in Ghana [22]. The districts are bordered by Burkina Faso to the north. In 2012, the Kassena-Nankana districts had a population of approximately 185,000 people [23]. The two major ethnic groups are the Kassenas and the Nankanis [24].

The ecological characteristic of the study area is that of Sahel with semi-arid Guinea savannah vegetation. There is one rainy season each year from June to October. The local economy depends on subsistence agriculture. The major crops cultivated in the area are millet, maize, sorghum and rice [21]. The majority of the people live in rural settings and households are grouped into extended family units or compounds [25]. The Kassena-Nankana districts are served by one hospital, six health centres, one private clinic and two mission clinics. Moreover, there are several private chemists, drug vendors, traditional healers and traditional birth attendants. Finally, the Navrongo Health Research Centre (NHRC) is also situated in the area, which has a good laboratory and runs a Health and Demographic Surveillance System (HDSS) [21, 24, 26]. Malaria, gastroenteritis and acute respiratory infection are the main causes of morbidity and mortality in the study area, and outbreaks of meningitis also occur periodically [21].

### Surveillance procedures

**Fig. 4:**
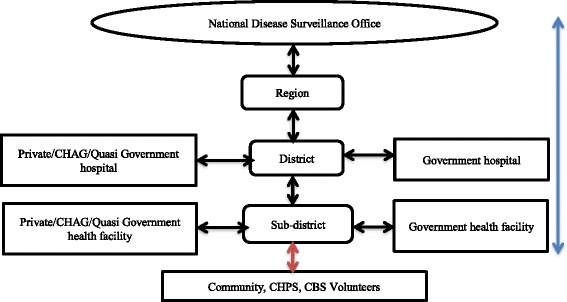
Flow of IDSR data in Ghana. Source: Ghana IDSR technical guidelines [16]

At the community level, surveillance activities are undertaken by local volunteers who are trained to observe and report diseases to the peripheral health facilities using simple case definitions [16, 17]. For example, a simple case definition of cholera for community surveillance is any person aged five years or over with lots of watery diarrhoea and sometimes vomiting profusely as well, while in case of cholera outbreak, any person who passes watery/loose stool is a suspected case [17]. Any person with fever and neck stiffness in the community is considered a suspected case of meningitis [17]. These simplified case definitions aim to enhance early detection of public health threats at the community level and prompt response from the health facility level. At the health facilities, the data are differentiated into out-patient, in-patient, consulting room and laboratory registers and transferred into daily summary sheets by the disease control officers. The data of the summary sheets are then entered into the IDSR reporting forms and sent to the District Health Directorate (DHD) as weekly, monthly or quarterly reports. The IDSR reports are received at the DHD by the district disease control officer or health information officer who enters the data from the paper-based forms into the DHIMS2 [16]. The information includes suspected cases, laboratory confirmed cases and deaths [20, 27]. Disease surveillance data analysis is required at all levels of the health system to determine trends and appropriate interpretation for effective response [16, 17]. Routinely, graphical presentations of the analyzed data are posted on the notice boards for public health education within the communities (Fig. 4).

### Routine diagnostic procedures

The standard case definitions in the IDSR guidelines are a set of criteria used to decide if a person has a particular disease or condition [16]. There are however, several diseases with similar signs and symptoms. Thus, biological specimens are required to be collected, stored and processed to achieve specific diagnoses (e.g. malaria) [16]. For suspected diseases which a periphery health facility lacks the capacity to perform laboratory tests for confirmation, specimens are sent to the district hospital or the district health directorate for onward delivery at a designated reference laboratory (e.g. tuberculosis, meningitis). The specimens are transported from health facilities using motor bikes or pick-up vehicles where applicable. At the district level, the specimen is transported by means of pick-up vehicle or motorbike while at the regional level specimens are transported mainly through the commercial transport system to the reference laboratories (e.g. Tamale Public Health Laboratory). At the periphery or district level, the disease surveillance officer or laboratory focal person is responsible for sending specimens to the reference laboratories. When specimens are sent to the reference laboratories, information on the name and address of the health facility as well as the name and telephone number (and e-mail address if available) of the focal person for surveillance are required for communication [16]. The important referral laboratories in the country are the Noguchi Memorial Institute for Medical Research and the National Public Health Reference Laboratories in Accra (e.g. Polio, Ebola), and the laboratories of regional hospitals in Accra, Sekondi, Kumasi, Koforidua, Sunyani, Tamale, Bolgatanga and Wa (e.g. cholera).

### Study design

A qualitative study design was applied to query key informants who are responsible for health care delivery at the periphery of the health system. Interviews were conducted on the core and support functions of the system using a semi-structured questionnaire.

### Sampling procedure

The UER is one of the three northern regions of Ghana. The region is remote from the national capital and has a higher likelihood of infectious disease epidemics. The Kassena-Nankana districts were selected for convenience to investigate the surveillance practice in more details. Within the two districts, we included in the study the only referral hospital (War Memorial Hospital), all the five health centres (Kologo, Navrongo, Kandiga, Paga & Chiana health centres), the only two mission clinics (St. Martin’s, Biu & Martyrs of Uganda, Sirigu) and the only private clinic (St. Jude clinic).

At the health facility, the medical officer or medical/physician assistant, public health nurse, disease control, laboratory and health information officers were qualified to participate in the study. The medical officer or medical/physician assistants are responsible for generating surveillance information using clinical diagnosis for suspected cases. This information is recorded in the consulting room register such as age, sex, and provisional diagnosis. The suspected case is then referred to the laboratory for further investigation and confirmation. After the samples from suspected cases have been tested in the laboratory, the medical officer or medical assistant updates the consulting room register with the information of principal diagnosis and/or additional diagnosis. On the other hand, the laboratory staff functions to confirm the provisional diagnosis as well as to provide information on any additional diagnosis after samples of suspected cases have been tested and determined according to standard case definition. While the disease control officer collates daily, the number of suspected cases, confirmed cases and deaths using the consulting room registers, laboratory registers and in-patient registers as sources of data. This information is submitted to the district health directorate using the weekly and monthly IDSR reporting forms. They also conduct case and contact tracing in the communities especially for epidemic prone diseases. The health information officer is responsible for the weekly and monthly IDSR data entry into DHIMS2. They are also responsible for analyzing the surveillance data and determining trends in disease occurrence. The public health nurse is responsible for health education and promotion campaigns during immunizations and disease outbreaks.

Eligibility for key informant participation included: a) working with the periphery health facility; b) familiarity and active involvement in public health, disease surveillance or health information activities; c) willingness to participate in the study; and d) completion of written consent. On the day of the field visit, the head of the health facility selected any two of the above list of health workers and the questionnaire was administered to them. However, new employees (less than three months at position) as well as respondents who demonstrated inadequate knowledge and non-involvement in disease surveillance activities at the health facility level were excluded.

### Study procedure and data collection

A total of 18 key informant interviews among health workers from nine health facilities were conducted. Two informants were interviewed per health facility using a semi-structured questionnaire. The main issues addressed in the questionnaire were: a) background information of key informants (e.g. job title/position, sex, education, number of years of work); b) availability of standard case definitions; c) core functions of the surveillance system (e.g. case detection/identification and registration, confirmation, reporting, analysis, feedback, and preparedness and response); d) support functions of the surveillance system such as training of health workers, supervision and resources for effective functioning and e) satisfaction with surveillance work. Examples of the specific questions which were asked included: “What are the challenges associated with identifying and recording of suspected disease cases for surveillance?” “What problems has this health facility had with handling and transporting of laboratory specimens to the next level for further investigation?” The informants were selected among the following disciplines present on the day of the field visit: head of health facility (medical/physician assistant), public health nurse, and specific officers for disease surveillance, health information and laboratory. Three of the respondents were subsequently replaced due to their insufficient knowledge on disease surveillance. The fieldwork was conducted between July and November 2013.

### Data analysis

The data was read multiple times, then transcribed and coded into the various themes of the core and support functions of the IDSR system. Broadly, the themes encompassed case detection and registration, case confirmation, data reporting, data analysis, epidemic response, feedback, supervision, training and resources.

### Limitations of study design

The study was conducted among health workers, thus, the fear of victimization from higher authority was likely to affect their answers. Moreover, the study design was limited in terms of determining trends and differences between health facilities. In order to remove such fears, the respondents were assured of anonymity and confidentiality. The strength of the design included detail information about the characteristics and perceptions (e.g. needs and desires) of the health workers regarding the IDSR system. It also created flexibility for the respondents to describe the IDSR system according to their own experiences and expectations.

### Ethical considerations

Individual written informed consent was obtained before the interviews were conducted. Ethical approval for the study was obtained from the Navrongo Health Research Centre Institutional Review Board (NHRCIRB155) and the Ethics Commission of the Heidelberg Medical Faculty (S-215/2013). Permission was also obtained from the Ghana Health Service in the region. The interviews were conducted in English by the first author.

## Results

The majority of the informants interviewed were males (12/18). Seven respondents were disease control officers, four were physician/medical assistants, three were general staff nurses, one was a community health nurse, one a biomedical scientist, one a health information officer, and one a nutrition officer. Over half (10/18) of the informants indicated that they were working at the same health facility for the past four or more years while 6/18 were working at this place for a period of one to three years and the remaining 2/18 less than a year.

### Overall functioning of the surveillance system

The informants had mixed views on the functioning of the IDSR system. Only a few (3/18) respondents said that the DHIMS2 implementation in 2012 contributed to improved disease surveillance. Nearly all respondents (17/18) reported that they were not satisfied with the disease surveillance performance. Reasons included lack of community member’s cooperation (e.g. delays in presentation of patients at facilities, refusal of referral for diagnostic procedures, low compliance with treatment), and inadequate staff for surveillance.

“*The community members do not cooperate with health staff in disease surveillance investigation. For example, tuberculosis cases do not comply with its treatment when given to patients. HIV* (Human Immunodeficiency Virus) *cases also refuse to go to War Memorial Hospital whenever they are referred for further laboratory test to confirm the diagnosis.”* Medical Assistant, Informant # 2

*“There is inadequate staff for disease surveillance and sometimes, suspected cases delay in the communities before visiting a health facility for diagnosis and treatment which affects early detection.”* Disease control, Informant # 7

### Case detection and registration

None of the 9 health facilities had copies of the national IDSR technical guidelines (2002 and 2011 editions) which contained the standard case definitions for surveillance. The majority (15/18) of the respondents agreed that health facilities had problems concerning suspected cases identification and recording. The main problems included limited laboratory capacity, discrepancies in laboratory diagnosis, perception of false results (e.g. tuberculosis diagnosis), unstable power supply, and poor recording of cases in the registers.

“*The health facility does not have adequate reagents for laboratory test and does not have the capacity to perform lumbar puncture. Unstable power supply to power the laboratory equipment affects case identification.”* Medical Assistant, Informant # 7

“*The identification of the suspected cases is not a major problem of the hospital, however, suspected cases are poorly recorded in the registers. There is often discrepancy between the recorded cases in outpatient registers and laboratory registers of health facility. For example, a suspected case may be diagnosed to be measles on Monday and then, on Tuesday, the records will be changed to rather reflect meningitis which makes it difficult for classification, recording, tallying and reporting to IDSR system.”* Disease Control, Informant # 9

“*The capacity of the laboratory is limited and unable to test most diseases or conditions. For example, the tuberculosis results from the laboratory are usually inaccurate. Suspected cases refused to return for further investigations on potential diseases. Some suspected cases are also difficult to confirm. For example, Yaws is difficult to be confirmed from the flipcharts.”* General Staff Nurse, Informant #15

### Case confirmation

All nine health facilities had the capacity to process stool samples for further investigation. Most (8/9) health facilities had the capacity to also process blood/serum for case investigation. There were, however, a number of challenges on further investigation and confirmation of suspected cases. These included unwillingness of patients to provide specimens for diagnosis confirmation, lack of transport and staff for bringing samples to the reference laboratories, limited capacity in laboratories for blood/stool storage, poor quality of specimens, and difficulties in tracing specimens at reference laboratories.

“We *lack transport to send specimens to district or regional hospitals for confirmation. Sometimes the laboratory staff takes poor quality specimens from suspected cases. One of our problems is that the suspected cases are unwilling to provide specimens for further investigation.”* Medical Assistant, Informant # 12

*“There is inadequate funding to transport specimens to reference laboratories, inadequate staff and no personnel responsible to travel with specimens to reference laboratories in Tamale or Accra. Missing personal details of specimen at the reference laboratory creates difficulties in specimen tracing.”* Biomedical Scientist, Informant # 10

### Data reporting

Mainly paper-based IDSR data was sent from the health facilities to the DHD, but sometimes information was also transmitted by mobile phone. Other challenges included shortage of IDSR reporting forms, irregular reporting, lack of regular access to consulting room registers, inadequate staff, overburdening of staff and inadequate funding.

*“The monthly and weekly IDSR forms are available. Whenever there are shortages, photocopies are made at the district health administration to replace. Sometimes, the internally generated funds are also used to make the photocopies.”* Medical Assistant, Informant # 2

*“The weekly and monthly IDSR reports are prepared and sent regularly. Whenever we failed to submit the reports on time, sub-district and district staff calls on phone to remind us and demand that the reports be submitted.”* Physician Assistant, Informant # 3

*“I don’t easily have access to the consulting room registers to count and tally suspected cases. This increases the waiting time and affects timely submission of IDSR reports to the district health administration.”* Disease control, Informant # 18

*“The staff responsible for IDSR activities is inadequate. An assistant is needed to improve reporting of IDSR data. The counting of the suspected cases from the consulting room and laboratory registers is so huge thereby overburdening the disease control officer.”* Disease control, Informant # 5

### Data analysis

Half (9/18) of those interviewed indicated that disease surveillance data was analyzed at the health facility level while the remaining reported that the data was not analyzed. However, if analyzed, the data analysis was often limited to the immediate notifiable diseases and to immunization coverage. Several reasons were cited for the failure to conduct comprehensive analysis of disease surveillance data such as inadequate personnel, and lack of recognition for surveillance activities.

“*The disease surveillance data has been analyzed on the immediate notifiable diseases and immunization coverage. You can see the graphs posted on our notice board in the yard. Sometimes, the graphs are quickly removed and replaced by other posters. The removal of the graphs has made me to stop performing analysis on the immediate notifiable diseases.”* Disease control, Informant # 5

“*The hospital does not recognize the disease control unit. This demotivates me from doing analysis on surveillance data. But we make analysis for the half-year reports and review meetings at the district health directorate.”* Disease control, Informant # 9

“*I am only acting as the disease control officer and I do not have adequate knowledge on the IDSR reporting and analysis of surveillance data.”* Nutrition officer, Informant # 17

### Epidemic response

Nearly all (17/18) the informants indicated that when epidemic prone diseases are suspected at the health facility level, there is some response such as specimen taking and reporting to the relevant authorities in the health system for appropriate public health action.

*“The immediate action we take includes the involvement of the community members on the suspected case such as polio, cholera and meningitis. In addition, district health administration is immediately notified of the suspected disease for immediate response.”* Disease control, Informant # 1

*“The case-based form is completed for the suspected case and the specimen is taken for investigation. The district health administration is notified immediately and the suspected case is also referred to the hospital for investigations.”* Disease control, Informant # 13

### Surveillance feedback

Nearly all the respondents (17/18) reported that no real feedback to the periphery level exists (e.g. written report or bulletin). Apart from direct investigations by phone in case of inconsistencies in the reports, feedback on disease surveillance to the health facilities only took place during the monthly unit head meetings or during the bi-annual or annual review meetings. In contrast, the health facilities provided feedback to their communities during durbars (formal community meetings,) and during health education talks at out-patient departments. The durbars are planned according to community events (e.g. annual festivals, launching of a new health centre, handing over of a new borehole).

“*The health facility receives disease surveillance feedback during the half year or annual review meetings. Normally, when the district or region sees discrepancies with the data, then they give feedback especially on delayed reporting. However, the staffs responsible for the DHMIS2 network communicate more often on the performance of the health facility to clarify issues based on the discrepancies as seen in the reports.”* Disease control, Informant # 1

“*Oral feedback is given to the medical assistant on visits to the district health administration. Sometimes, the district disease surveillance officer calls on phone to clarify the IDSR data whenever discrepancies are detected in their IDSR reports.”* Medical Assistant, Informant # 12

“*Community durbars are the platforms where disease surveillance feedback is provided to the community members once or twice in a year per community (*e.g. *malaria and nutrition project supported by UNICEF –* United Nations Children’s Fund*).”* Disease control, Informant # 15

### Surveillance supervision

The majority of the respondents (15/18) indicated that the health facilities have been supervised by officials of the district or regional health directorates. However, they reported that such visits were irregular and also not purposely for disease surveillance except during epidemics. One particular informant was uncertain whether supervision ever occurred for the purpose of disease surveillance in the absence of outbreaks. Community surveillance volunteers were sometimes supervised by the staff of a peripheral health centre according to an existing plan.

*“The health facility has been visited by district and regional officials for supervision. We have a visitor’s book where the supervisors write their names, name of organization/unit and date of visit including the signature. Many different units and departments come together on supervision but they do not come separately for disease surveillance.”* Disease control*,* Informant # 1

*“I don’t know if there was supervisory visit by higher officials for disease surveillance activities. I remember that HIV/AIDS* (Human Immunodeficiency Virus/Acquired Immune Deficiency Syndrome) *team came recently on supervisory visit to monitor such cases at the hospital.”* Biomedical scientist*,* Informant # 10

*“The health facility has supervised the community-based surveillance volunteers. There is a plan for visiting the community-based surveillance volunteers and the dates for the visits are known by the volunteers in advance.”* Disease control*,* Informant # 8

### Surveillance training

Only a minority of the respondents (2/18) had university degree qualification. Nearly all (8/9) of the health facilities visited had a trained disease control officer. However, they continued to receive refresher training while on the job. The beneficiaries of such disease surveillance trainings have however failed to multiply the knowledge in the health facilities by training other staff. Various reasons were cited for this failure such as their own lack of in-depth knowledge and an overall lack of interest and recognition for disease surveillance.

*“In 2009 and 2011, I was trained on disease surveillance during my nursing course and I have also been trained after school by Ghana Health Service.*” General staff nurse, Informant # 6

*“Disease surveillance is considered a unit issue which has no impact on the hospital performance. The laboratory is not involved in disease surveillance. I must be honest; we at the laboratory do not know the alert and action thresholds for epidemic prone diseases.*” Biomedical scientist, Informant # 10

*“We do not train other staff at the health facility because they do not have interest in public health and disease surveillance activities. Disease surveillance is considered a boring task by the staff. It is perceived to be distraction to the work of prescribers/clinicians since they use the same consulting room registers for their work too.*” Disease control, Informant # 16

*“We cannot train other staff because of inadequate funds, lack of interest by nurses and refusal of staff to be trained. For example, community health nurses refused to be trained on surveillance. Unfortunately, one of these same nurses is now in-charge of a health facility and has to prepare both the weekly and monthly IDSR reports alone.*” Disease control, Informant # 18

### Surveillance resources

According to the respondents, resources for surveillance were inadequate in terms of financial, human, infrastructure and material. Also transfer and turnover of the staff had negative effects on the functioning of the IDSR system.

*“As you can see, the disease control unit does not have a sitting place at the hospital to do surveillance activities. We are only sharing the child and maternity ward at the hospital as disease control office. We have no means of transport (*i.e. *no motor bike and no vehicle). Although, there are many vehicles at the hospital, not a single one is allocated for disease surveillance purpose.”* Disease control*,* Informant #9

*“The health facility does not even have one single phone to report disease surveillance information except our personal mobile phones.”* General staff nurse*,* Informant # 15

## Discussion

This study provides an overview of the quality of the core and support functions of the Integrated Disease Surveillance and Response (IDSR) system in northern Ghana. The adoption and implementation of the IDSR strategy over a decade ago has shown some improvements in disease surveillance activities in several countries which include a potentially more rapid dissemination of information and the inclusion of new diseases such as non-communicable diseases into the system [8, 9]. Results from this study in Ghana show that the IDSR strategy has contributed to increased surveillance report submission. It has also partly contributed to enhance analysis of surveillance data at the periphery health facilities. In addition, the IDSR strategy enabled each health centre to have a designated disease surveillance officer. However, the main finding from this study is that despite the adoption of the IDSR strategy, disease surveillance remains a neglected area in Ghana. This is similar to findings from other Sub-Saharan Africa (SSA) countries which reported very limited capacity for disease surveillance [9, 15, 28, 29].

### Core functions

The study revealed weaknesses in case identification and recording at the periphery of the Ghana health system, which supports similar reports from other SSA countries [9, 11, 30]. The consulting room registers for capturing patient’s personal information are rather detailed, which contributes to mistakes in data recording and to overburdening of staff. For instance, clinicians have three diagnostic categories for each suspected case i.e. provisional, principal and additional diagnosis. The likelihood not to put data into the principal and additional diagnosis columns was frequently observed in this study. The study also revealed difficulties in the confirmation of clinically suspected cases due to transport and compliance problems (e.g. HIV/AIDS and Tuberculosis) in addition to overall very limited laboratory capacity. The reported unwillingness of patients to provide specimens and their perception, that diagnostic testing at the periphery laboratories is not reliable, likely further affects the completeness of disease surveillance. Despite very visible and well supported national disease-specific programs (e.g. Tuberculosis and HIV), appropriate implementation of all components remains a challenge at the periphery of the health system. Thus, the possibility of missing outbreaks due to the obvious lack of confidence of the population appears to be real. This is evidenced in the recent outbreak of Ebola in West Africa, where many of the cases were not identified or confirmed on time [31]. Better public information and communication on surveillance aspects is urgently needed for an appropriate community involvement.

It was repeatedly reported by the informants that forms for IDSR were not available, which likely contributes to missing reports and incomplete data on specific diseases. Moreover, it limits the ability of the district and regional levels to adequately conduct data verification and validation. To address this problem, health workers sometimes have to use their personal funds or internally generated funds to make photocopies. Similar findings of unavailability of IDSR reporting forms and use of personal resources has been reported from other developing countries [32]. However, the reported initiatives of the health staff to solve these problems can be considered positive.

Electronic reporting on District Health Information Management System II (DHIMS2) and mobile phone texting or phone calls are additional strategies adopted by the health system to ensure that disease surveillance data arrive at the district. Although periphery facilities do not yet directly enter data into the DHIMS2 (with the exception of the district hospital), the informants were of the opinion that it has the potential to improve timely submission and continuous access to reported data. With the electronic system, IDSR data can in principle continuously be monitored, verified and evaluated by the supervisory units of the health system to provide reliable and timely information for public health action. This would also reduce the travel frequency between the health facilities and district health directorate for IDSR reports submission. In addition, with the in-built data analysis capability of DHIMS2, the health facilities can in principle perform surveillance data analysis for comparison, early warning and rapid response. This is in line with multiple reports from the developing world that components of m-Health have a huge capacity to improve the efficacy of health systems [30, 33].

The capacity for data analysis and interpretation has been shown in this study to be rather limited at the periphery of the Ghana health system. While the immediate notifiable diseases as well as immunization coverage are at least partly considered, no further detailed analysis takes place, which has clear implications for potential contact tracing. Thus, the utilization of surveillance data for planning and decision-making at the periphery level remains very low [8]. However, there is some evidence for an increase in the utilization of surveillance data in recent years at Ghana district, regional and national levels [9].

The peripheral health system remains the first level of contact with the population for suspected cases and for surveillance data production. However, feedback to periphery facilities is still rare and irregular in most of SSA except during epidemics [9, 11, 30]. Disease surveillance feedback has been described as one of the most important activities for improving health workers capacities and performance [28]. On the other hand, this study shows that the Ghanaian health workers in the periphery have successfully used community gatherings to disseminate specific health information to the population.

### Support functions

The findings from this study demonstrate that supervision for surveillance at the periphery of the Ghana health system is rather poor and inadequate, which supports previous findings [8, 9]. Although there is some improvement in the deployment of disease surveillance officers and nurses to the periphery levels, the level of training is frequently not adequate for effective and efficient disease surveillance.

The respondents confirmed the existence of global problems such as inadequate staff, inadequate funding, frequent staff turnover and poorly equipped laboratories seriously affecting disease surveillance activities, which supports previous findings from Ghana [15]. Apart from insufficient human resources, problems with funding and material support are among the major factors contributing to poor surveillance data quality [32].

The study has some limitations. Firstly, as the investigations were limited to a small area in the North, findings are not representative of the entire health system in Ghana. Secondly, most of the information was self-reported and is thus likely influenced by individual factors of the limited number of respondents.

## Conclusion

In conclusion, although the IDSR system was associated with some benefits to the health system such as tracing of delayed reports and better availability and accessibility of electronic reports, there remain major challenges to the functioning and the quality of DHIMS2 in Ghana. As disease surveillance has obviously remained a neglected area of health systems in SSA, increasing attention to and recognition of surveillance activities as essential for the overall functioning of the health system are urgently needed. With the event of the dramatic and ongoing Ebola epidemic in West Africa, this becomes even more evident for early detection and appropriate response to prevent outbreaks and spread.

## Abbreviations

CBSV: Community-Based Surveillance Volunteers
CHPS: Community-based Health Planning and Services
DHD: District Health Directorate
DHIMS2: District Health Information Management System II
HDSS: Health and Demographic Surveillance System
HIV/AIDS: Human Immunodeficiency Virus/Acquired Immune Deficiency Syndrome
IDSR: Integrated Disease Surveillance and Response
IHR: International Health Regulations
NHRC: Navrongo Health Research Centre
NHRCIRB: Navrongo Heath Research Centre Institutional Review Board
PHC: Primary Health Care
SSA: Sub-Saharan Africa
TB: Tuberculosis
UER: Upper East Region
UNICEF: United Nations Children’s Fund
WHO: World Health Organization
WHO-AFRO: World Health Organization Regional Office for Africa
